# Exploring the effect of tandem non-native non-F-type lectin carbohydrate-binding domains on *Streptosporangium roseum* α-L-Fucosidase activity

**DOI:** 10.1016/j.bbrep.2026.102797

**Published:** 2026-09-15

**Authors:** Shivangi, Madhu Lata, Srikrishna Subramanian, T.N.C. Ramya

**Affiliations:** a CSIR- Institute of Microbial Technology, Sector 39-A, Chandigarh, 160036, India; b Academy of Scientific & Innovative Research (AcSIR), Ghaziabad, Uttar Pradesh, 201002, India

**Keywords:** α-L-fucosidase, Carbohydrate-binding domain, CBM, Lectin, Tandem domain

## Abstract

Microbial carbohydrate-active enzymes often contain non-catalytic carbohydrate-binding modules, which enhance the activity of the catalytic domain by proximity, targeting, or disruptive effects. We have previously biochemically characterized a GH29 α-L-fucosidase from *Streptosporangium roseum,* demonstrated that its C-terminal F-type lectin domain (FLD) improves the enzyme activity of its tandem N-terminal α-L-fucosidase domain towards fucooligosaccharides, and proposed a “bind and jump” model to explain this FLD-driven improvement of α-L-fucosidase activity. Here, we have explored the use of non-native, non-FLD carbohydrate-binding domains to similarly enhance the enzymatic activity of the α-L-fucosidase domain. We employed three carbohydrate-binding domains (MG1D1, MN3α, and MU1α) that we previously identified from the gut microbial metagenome by functional screening, and fused them to the N-terminus of the *S. roseum* α-L-fucosidase domain. The constructs showed activity on a synthetic substrate, 4-Methylumbelliferyl-α-L-fucopyranoside. However, when assaying these constructs on the natural fucosylated oligosaccharides, Lewis a tetrasaccharide and 2′-fucosyllactose, we did not observe a statistically significant improvement in the α-L-fucosidase activity of MN3α-*Sr*Fuc, MG1D1-*Sr*Fuc, or MU1α-*Sr*Fuc, compared to the *Sr*Fuc construct containing only the α-L-fucosidase domain. Our results indicate that the tandem positioning of these non-native, non-FLD carbohydrate-binding domains does not significantly improve the α-L-fucosidase activity of *Sr*Fuc towards these oligosaccharides. Additional chimeric constructs with domain organizations designed to closely mimic the wild-type protein *Sr*FucNaFLD, and further studies focused on the mechanistic basis of the FLD-mediated improvement of α-L-fucosidase activity, might help translate the strategy and design chimeric α-L-fucosidases with non-native, non-FLD carbohydrate-binding domains that exhibit improved α-L-fucosidase activity towards these oligosaccharides.

## Highlights

• *Sr*FucNaFLD has an α-L-fucosidase domain (Fuc) and an F-type lectin domain (FLD).
• The tandem FLD is known to increase α-L-fucosidase activity of *Sr*Fuc.
• We assessed the effect of tandem non-native non-FLD domains on the α-L-fucosidase.
• We used terminal LacNAc-binding non-native non-FLD domains MG1D1, MN3α, and MU1α.
• We did not note increased activity for Lewis a tetraose and 2′-fucosyllactose.

## 1 Introduction

Carbohydrate-active enzymes frequently contain a non-catalytic carbohydrate-binding module (CBM) in addition to the enzyme domain. CBMs function via proximity, targeting, and disruptive effects, and improve the enzyme activity of the tandem catalytic domain by increasing access to the substrate [[1], [2], [3]].

We previously biochemically characterized an α-L-fucosidase (*Sr*FucNaFLD) with an N-terminal α-L-fucosidase domain (Abramson, J., Adler, J. et al.), a central NPCBM-associated (Na) domain, and a C-terminal CBM47/F-type lectin domain (FLD) from *Streptosporangium roseum.* We demonstrated that the tandem presence of the FLD improves the enzymatic activity of the α-L-fucosidase domain towards aqueous fucooligosaccharide substrates and proposed a “bind and jump” model to rationalize this observation [4]. This model envisages that the fucooligosaccharide initially docks in the binding pocket of the FLD and then jumps into the active site of the α-L-fucosidase domain, whereupon hydrolysis occurs, and the released L-fucose jumps into the binding pocket of the FLD (thereby reducing product inhibition) and is displaced by a fucosylated oligosaccharide substrate molecule (owing to the lower affinity for L-fucose). We observed that the Na domain is expendable, and the native FLD can be swapped with a non-native FLD from *Actinomyces turicensis* without loss of FLD-mediated improvement of α-L-fucosidase activity [5].

Building on this study, we have now asked the question whether the tandem presence of non-native, non-FLD carbohydrate-binding domains can similarly enhance the enzyme activity of the α-L-fucosidase domain of *S. roseum* (*Sr*Fuc). We selected the carbohydrate-binding domains (MG1D1, MN3α, and MU1α) of three mucin-binding proteins (MG1, MN3, and MU1), which we previously identified through a functional screen of a metagenome-derived phage display library constructed from the fecal DNA of healthy human subjects, and determined to bind to N-glycans with terminal N-acetyllactosamine units [6].

We felt that the study would be interesting from the perspective of the oligosaccharide motif on the substrate recognized by the carbohydrate-binding and catalytic domains. Whereas in *Sr*FucNaFLD, both domains *Sr*Fuc and *Sr*FLD bind to the same terminal α-L-fucose moiety, requiring a “bind and jump” event, in chimeric constructs of *Sr*Fuc fused with these carbohydrate-binding domains (MG1D1, MN3α, and MU1α, which primarily bind to LacNAc/Lac), a “bind and jump” model might not be a strict requirement if simultaneous binding to the respective motifs by the two domains is sterically feasible.

However, employing chimeric constructs containing *Sr*Fuc in tandem with these carbohydrate-binding domains, we found that the tandem presence of the non-native non-FLD carbohydrate-binding domains, MG1D1, MN3α, or MU1α, in *Sr*Fuc did not result in improvement of α-L-fucosidase activity towards Lewis a tetrasaccharide and 2′-fucosyllactose.

## 2 Results

### 2.1 Design of carbohydrate-binding domain fusion constructs with *S. roseum* α-l-fucosidase

The full-length α-L-fucosidase protein from *Streptosporangium roseum* [4], *Sr*FucNaFLD, comprises, in N-to-C-terminal order, a signal peptide, a retaining-type GH29 α-l-fucosidase domain (PF01120) with an α-L-fucosidase_C domain (PF16757), a NPCBM-associated (Na; NEW3 domain; PF10633) domain, and an F-type lectin domain (FLD; PF00754). Previously, we have demonstrated that the tandem presence of the FLD enhances α-L-fucosidase activity on fucooligosaccharides [4,5], an effect we attributed to a “bind and jump” (or “internal diffusion” or “substrate channeling”) mechanism [4,5]. We also recently demonstrated that a chimeric protein, comprising the *Sr*Fuc and Na domains, and a non-native FLD from *Actinomyces turicensis*, showed enhanced α-l-fucosidase activity, compared to just the *Sr*Fuc construct [5].

**Fig. 1:**
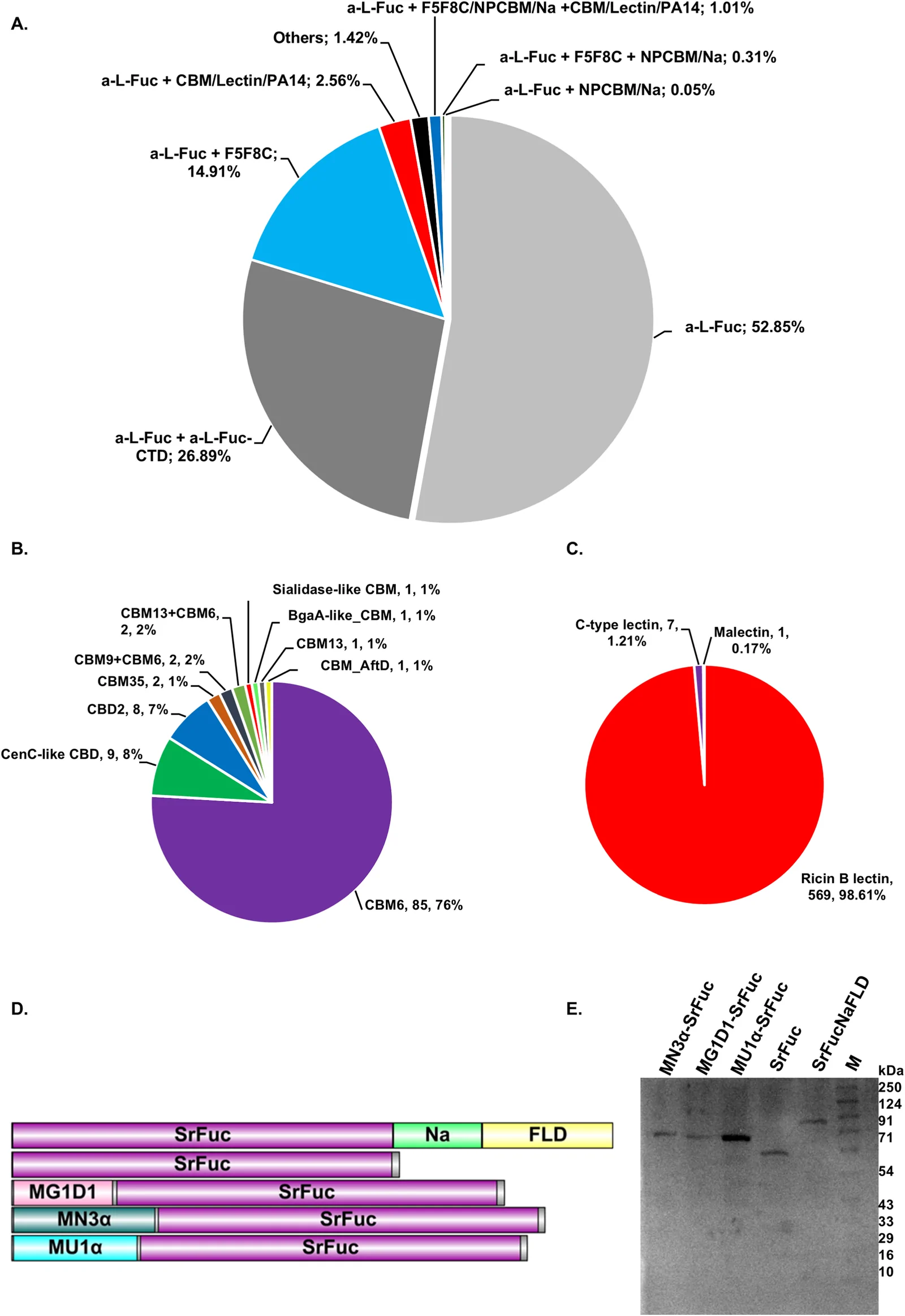
**Domain architecture analysis and α-L-fucosidase constructs employed in the study**. (**A**) Distribution of domain architectures of proteins containing a GH29 α-L-fucosidase domain (PF01120). (**B**) Distribution of Carbohydrate-Binding Modules (CBMs) co-occurring with the α-L-fucosidase domain (PF01120). (**C**) Distribution of lectin domains co-occurring with the α-L-fucosidase domain (PF01120). (**D**) Domain architecture of constructs used in the study. The domain architectures were made using Illustrator of Biological Sequences (IBS) [8]. (**E**) Coomassie blue-stained SDS-polyacrylamide gel showing purified recombinant proteins of the constructs used in the study. M: Puregene™ protein marker.

Analyzing the domain architectures of the PF01120 α-L-fucosidases in the InterPro database [7] indicates that the tandem domains found in *Sr*FucNaFLD are also prevalent in other proteins with the PF01120 α-L-fucosidase domain. The Interpro database (as of 25 December 2025) comprised 435 unique domain architectures and 24,338 protein sequences containing the PF01120 α-L-fucosidase domain (Fig. 1A, Table S1). Among them are 12,863 standalone α-L-fucosidase domains (PF01120), accounting for a little more than 50% (Fig. 1A–Table S1). Roughly over a quarter of the PF01120-containing proteins also contain a fucosidase_C domain (PF16757), as in *Sr*FucNaFLD (Fig. 1A–Table S1). There is also a significant number of proteins with F5F8 domains (PF00754/F5_F8_type_C, which includes FLDs, and PF22633/F5_F8_type_C_2) and NPCBM/NPCBM-associated domains (PF08305/PF10633) (Fig. 1A–Table S1). Interestingly, ∼4% of the proteins contain carbohydrate-binding domains, including non-FLD CBMs and lectin domains, and PA14 domains (Fig. 1A–Table S1). We observed that the CBMs co-occurring with the PF01120 α-L-fucosidase domain include CBM6, CBM9, CBM13, CBD2, CenC-like CBD, sialidase-like CBM, CBM-AftD, BgaA-like CBM, and CBM35, and that the lectin domains include ricin B lectins, C-type lectins, and malectin (Fig. 1B and C, Table S2 and S3). These prevalent domain architectures suggest that the α-L-fucosidase activity of the PF01120 domain might also be modulated by non-FLD carbohydrate-binding domains. Accordingly, we designed three α-L-fucosidase chimeric constructs in this study that contain non-native non-FLD carbohydrate-binding domains. We employed the novel carbohydrate-binding domains (the ricin B-like β-trefoil domain MG1D1, and the β-sandwich domains MN3α and MU1α) of the previously reported mucin-binding proteins, MG1, MN3, and MU1, identified by biopanning a phage display library constructed from metagenomic DNA from the feces of healthy individuals against mucin [6]. The carbohydrate-binding domains from these clones were used to generate the α-L-fucosidase chimeric constructs, MG1D1-*Sr*Fuc, MN3α-*Sr*Fuc, and MU1α-*Sr*Fuc (Fig. 1D–S1, Table S4), which were employed in this study alongside the previously reported *Sr*Fuc and *Sr*FucNaFLD constructs (Fig. 1D).

We have previously described the AlphaFold3 [9]-predicted structural models of *Sr*Fuc and *Sr*FucNaFLD [5]. We obtained high-confidence predictions of the structural models of MG1D1-*Sr*Fuc, MN3α-*Sr*Fuc, and MU1α-*Sr*Fuc using the AlphaFold3 webserver (average pLDDT scores of 91.62, 91.61, and 93.00, respectively) (Fig. S2). The PAE plots indicated suboptimal confidence in the positioning of the MG1D1/MN3α/MU1α domain relative to the α-L-fucosidase domain in these constructs, suggesting a flexible linkage between the two domains (Fig. S2). Boltz2 [10]- predicted structural models of L-fucose complexes for all constructs (*Sr*Fuc, *Sr*FucNaFLD, MG1D1-*Sr*Fuc, MN3α-*Sr*Fuc, and MU1α-*Sr*Fuc; all 25 models for each construct) showed L-fucose in the α-L-fucosidase active site (Fig. S2), and the top-ranked models showed high binding probabilities (affinity_probability_binary >0.8) (Table S5). Structural alignments of the top-ranked models with the publicly available crystal structure of *Thermotoga maritima* α-L-fucosidase (PDB ID: 1ODU) indicated overall protein chain alignment with low RMSD values (Table S6), and a reasonable agreement in the L-fucose position and conformation, as indicated by the low static and fit RMSD values (Table S7). Our efforts to predict complexes of these proteins with the fucooligosaccharides, Lewis a tetrasaccharide and 2′-fucosyllactose, and their disaccharide components (Fucα1-2Gal and Fucα1-4GlcNAc) were not successful (affinity_probability_binary > 0.5), perhaps due to large ligand size or the nature of the ligand [10] (Table S5). The individual domains *Sr*FLD and MG1D1, but not MN3α or MU1α, were also predicted to bind L-fucose (affinity_probability_binary > 0.7) (Fig. S2, Table S5), suggesting stronger binding to L-fucose by the former domains.

All these recombinant proteins were successfully expressed and purified (Fig. 1E, Fig. S3). We noted a mutation, A57D (relative to the original MG1 clone), in the MG1D1 domain of the MG1D1-SrFuc clone (Fig. S1). Although the ligand-binding pocket of MG1D1 has not been experimentally determined, A57 is not in the vicinity of L-fucose in the Boltz2-predicted L-fucose-bound MG1D1 model, and hence the mutation A57D is unlikely to affect ligand binding. Analysis of their oligomeric status by DLS suggested that all the recombinant proteins existed as a mixture of a 7-9 mer and a >2000 kDa higher order oligomer or aggregate (Fig. S4), similar to what we had observed previously for *Sr*Fuc and its constructs [5], except for the absence of the small proportion of dimer that we had observed previously in *Sr*FucNaFLD.

### 2.2 Enzyme activity of carbohydrate-binding domain fusion constructs of *S. roseum* α-l-fucosidase

**Fig. 2:**
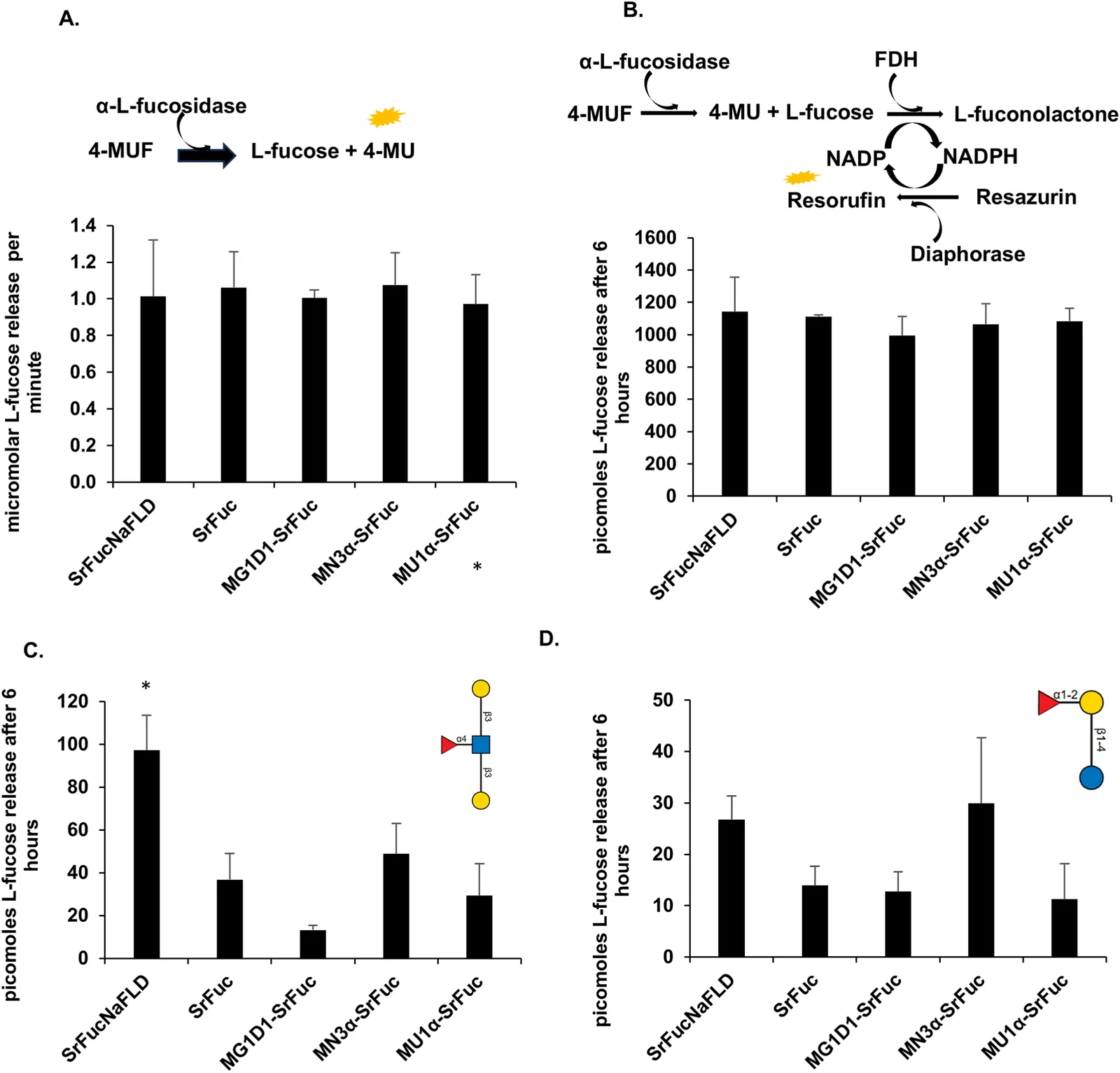
**Enzyme activity of α-L-fucosidase constructs employed in the study.** (**A**) Bar plot of initial velocities of α-L-fucosidase constructs for the synthetic substrate 4-MUF. The reaction mixtures contained 10 μM 4-MUF and α-L-fucosidase at concentrations adjusted to achieve normalized L-fucose release. The reaction scheme is presented above the bar plot. (**B**) Bar plot of L-fucose released by α-L-fucosidase constructs from the synthetic substrate 4-MUF (10 μM). The same reaction used in Fig. 2A was coupled with FDH and Diaphorase at the endpoint of 6 h. The reaction scheme of the coupled assay is presented above the bar plot (**C**) Bar plot of L-fucose released by α-L-fucosidase constructs from the natural fucosylated substrate Lewis a tetrasaccharide after 6 h, as measured by the FDH- and Diaphorase-coupled assay. The reaction mix contained 1 mM Lewis a tetrasaccharide and α-L-fucosidase at concentrations adjusted to achieve normalized l-fucose release from 4-MUF. (**D**) Bar plot of L-fucose released by α-L-fucosidase constructs from the natural fucosylated substrate 2′-fucosyllactose after 6 h, as measured by the FDH- and Diaphorase-coupled assay. The reaction mix contained 1 mM 2′-fucosyllactose and α-L-fucosidase at concentrations adjusted to achieve normalized l-fucose release from 4-MUF.

We first measured the activity of all these α-L-fucosidase constructs on 4-MUF (4-methylumbelliferyl alpha-L-fucopyranoside). Using an enzyme concentration of 0.3 μM, we observed significant variation in enzyme activity across batches towards the synthetic substrate (Fig. S5), as previously reported in our studies [4,5]. Given that such variation would pose a challenge to elucidating the effect of the carbohydrate-binding domain on α-L-fucosidase activity with natural fucosylated substrates, we adjusted protein concentrations (Fig. S5) to normalize activity across all biological replicates and constructs relative to the synthetic substrate 4-MUF. Reactions set up with these adjusted concentrations showed similar α-L-fucosidase activity for all constructs, as expected, as measured by 4-MU fluorescence in real time (Fig. 2A) and also by resorufin fluorescence in an FDH- and Diaphorase-coupled end-point assay after a 6-h incubation period (Fig. 2B).

We proceeded to assay α-L-fucosidase activity on the fucooligosaccharides, Lewis a tetrasaccharide, and 2′-fucosyllactose using the FDH- and Diaphorase-coupled end-point assay, with the aforementioned protein concentrations normalized for similar specific activity on the synthetic substrate 4-MUF (Fig. 2C and D). We measured the amounts of L-fucose released by the different constructs after the 6-h incubation period.

With Lewis a tetrasaccharide, *Sr*FucNaFLD displayed >2.5-fold higher L-fucose release than *Sr*Fuc (Fig. 2C), indicative of tandem FLD-mediated improvement of α-L-fucosidase activity [4,5]. Among the carbohydrate-binding domain fusion constructs, MG1D1-*Sr*Fuc displayed a >2.5-fold reduction in L-fucose release compared to *Sr*Fuc; MN3α-*Sr*Fuc and MU1α-*Sr*Fuc did not exhibit an appreciable increase or reduction in L-fucose release in comparison to *Sr*Fuc (Fig. 2C). Application of a one-way ANOVA test against *Sr*Fuc (considering a significance threshold of 0.05) indicated a statistically significant change in L-fucose release only for *Sr*FucNaFLD (with an adjusted p-value of 0.01 in the one-way ANOVA followed by Dunnett's multiple comparisons test).

With 2′-fucosyllactose, too, *Sr*FucNaFLD displayed ∼2-fold higher L-fucose release than *Sr*Fuc, MG1D1-*Sr*Fuc and MU1α-*Sr*Fuc displayed L-fucose release in the same range as *Sr*Fuc, and MN3α-*Sr*Fuc showed ∼2-fold higher L-fucose release in comparison to *Sr*Fuc (Fig. 2D). However, the application of a one-way ANOVA test against *Sr*Fuc (considering a significance threshold of 0.05) indicated no statistically significant change in L-fucose release (adjusted p-values >0.05 in the one-way ANOVA followed by Dunnett's multiple comparisons test).

The assays with natural fucosylated substrates thus indicated no significant improvement upon N-terminal tandem fusion of the non-native, non-FLD carbohydrate-binding domains, MG1D1, MN3α, and MU1α (Fig. 2C and D).

## 3 Discussion

Employing *Sr*FucNaFLD as a model CAZyme that exhibits FLD-driven improvement of α-L-fucosidase activity, we report here that the non-native non-FLD (β-sandwich) carbohydrate-binding domains MN3α, (ricin-like) MG1D1, and (β-sandwich) MU1α do not significantly improve the α-L-fucosidase activity of *Sr*Fuc for α1,2-linked (2′-fucosyllactose) or α1,4-linked (Lewis a tetraose) L-fucose-containing substrates upon tandem placement. Although we observed ∼2-fold higher L-fucose release from 2′-fucosyllactose by MN3α-*Sr*Fuc, a magnitude similar to that observed for *Sr*FucNaFLD, the increase was not statistically significant for either construct in a one-way ANOVA, perhaps due to greater variability.

MG1, MN3, and MU1 were isolated from a functional screen as mucin-binding proteins (MG1 eluted by D-galactose, GalNac, and sialic acid, MN3 eluted by D-mannose and L-fucose, and MU1 eluted by D-GalNac), and all three clones displayed binding to asialo complex N-glycans and α1,2-linked L-fucose (H antigen) motifs [6]. However, whereas MG1 and MU1 exhibited binding to long N-glycans on a glycan array, MN3 exhibited binding to short N-glycans on the array and binding to H type-2 triose and Lewis y tetraose in enzyme-linked lectin assays [6]. Both MN3 and MU1 also bound to H type-2 pentaose, but notably did not bind to H type-2 triose or Lewis a tetraose in isothermal calorimetry runs [6]. The binding of H-type 2 triose in the enzyme-linked lectin assay, but not in the ITC run, likely stemmed from the multivalent presentation format of the former assay and the weak affinity (in solution) of the latter.

The current study employed the carbohydrate-binding domains MG1D1, MN3α, and MU1α, which differ from MG1, MN3, and MU1 in containing only a single, complete carbohydrate-binding domain and lacking extraneous N- or C-terminal regions of the polypeptide that do not correspond to the carbohydrate-binding domain. The current study employed Lewis a tetraose (as in the previous study [6]) and 2′-fucosyllactose (which is similar to H type-2 triose used in Ref. [6], differing only in the presence of Glc as against GlcNAc at the reducing end). We expected that the carbohydrate-binding domains, especially MN3α, by virtue of its preference for short α1,2-linked (but not α1,4-linked) L-fucosylated oligosaccharides, would bind to and enable an improvement in the α-L-fucosidase activity of the tandemly positioned *Sr*Fuc domain for 2′-fucosyllactose (but not Lewis a tetraose).

A limitation of our study is that we were unable to determine the mechanistic basis of the observed results. The lack of improvement in the α-L-fucosidase activity of the tandemly positioned *Sr*Fuc domain could be due to the carbohydrate-binding domains MG1D1, MN3α, and MU1α lacking sufficient affinity for the substrate to mediate channeling. Factors other than substrate affinity that might be responsible for the variable effect mediated by MN3α, MN3α and MG1D1 include access to substrate, construct oligomerization (and resulting avidity), and protein stability. Whereas the PAE plots indicate flexibility between the two domains across all constructs, the binding-site locations in MG1D1, MN3α, and MU1α remain unknown; therefore, the accessibility of substrates is unclear.

Avidity is a prevalent strategy among carbohydrate-binding proteins, and we previously reported that engineered FLDs with increased oligomerization exhibit enhanced binding strength. Although our DLS measurements indicated overall similarity in the oligomeric profiles, we were unable to determine the exact oligomeric state of these proteins in this study. The size-exclusion chromatography fractionation range would not resolve oligomers larger than hexamers or heptamers for most of these constructs, and we were unable to perform analytical ultracentrifugation or multi-angle light scattering on these proteins.

CBMs and non-CBM domains have also been reported to impart additional structural stability to tandem CAZyme domains [11,12]. Unfortunately, thermal shift assays of the constructs employed here did not yield conclusive results; therefore, we cannot rule out the possibility that the constructs used in this study differ in stability. However, we did note differences in α-L-fucosidase activity toward the synthetic substrate, 4-MUF, likely due to differences in stability. Further assays on Lewis a tetraose and 2′-fucosyllactose involved normalized protein concentrations using the synthetic substrate (4-MUF) to establish an equivalent baseline catalytic activity (assuming that the F-type lectin domain and the appended CBMs do not contribute to substrate recognition or binding of the small synthetic substrate) so that any differences observed during hydrolysis of fuco-oligosaccharides were more likely to reflect the influence of the appended carbohydrate-binding domains rather than differences in the intrinsic catalytic activity of the enzyme preparations. It is possible that differential enzyme activity was missed due to the single-endpoint measurement employed after 6 hours to assess α-L-fucosidase activity, as endpoint measurements provide only a snapshot and cannot distinguish among effects on catalytic rate, substrate binding, relief of product inhibition, and enzyme stability over the incubation period.

Another limitation of our study is that the constructs contained an N-terminal carbohydrate-binding domain and a C-terminal α-L-fucosidase domain. This is in contrast to the wildtype protein, where the α-L-fucosidase is towards the N-terminus [4]. Better FLD-mediated improvement of α-L-fucosidase activity is observed in *Sr*FucNaFLD and *Sr*FucFLD than in the domain order mutants, *Sr*FLDNaFuc and *Sr*FLDFuc [5]. Domain architecture analysis of the α-L-fucosidase family (PF01120) in the InterPro database revealed that while ∼98% of the proteins in this family contain an N-terminal α-L-fucosidase domain, ∼40% of the domain architectures (accounting for the remaining ∼2% proteins) contain a central or C-terminal α-L-fucosidase domain. Importantly, 16 unique proteins in 12 distinct domain architectures contained a ricin or other lectin domain, a CBM, or a PA14 domain on the N-terminal side of the PF01120 domain (Table S8). Hence, it is possible that the domain order is important for non-native, non-FLD carbohydrate-binding domains as well. Future studies exploring the effect of C-terminal fusions of MN3α, MU1α, and MG1D1 to *Sr*Fuc might be fruitful.

In our recent study employing various *Sr*FucNaFLD constructs with non-native FLDs, only one non-native FLD (*At*FLD from *Actinomyces turicensis*) mediated improvement of α-L-fucosidase upon tandem placement [5]. Analysis of the native context of *At*FLD indicated that it, too, possessed a tandem GH29 α-L-fucosidase domain, and hence, perhaps, had co-evolved to perform this function, unlike the other FLDs employed in that study [5]. The MG1 protein sequence shares similarity with proteins containing a GH65 N-terminal domain, and the MN3 and MU1 sequences share similarity with lipocalin-like domain-containing proteins from *Prevotella* species [6]. There is no clear indication yet if the carbohydrate-binding domains used in the study, MG1D1, MN3α, and MU1α, indeed exist as CBMs and have co-evolved with any catalytic module. With the increasing inflow of sequencing data and improvements in sequence annotation, it may be possible to strategically select carbohydrate-binding domains for tandem positioning, thereby increasing the likelihood of enhancing α-L-fucosidase activity toward the desired substrates.

To conclude, the tandem positioning of the non-native, non-FLD carbohydrate-binding domains MG1D1, MN3α, and MU1α did not elicit an improvement in the α-L-fucosidase activity of *Sr*Fuc towards 2′-fucosyllactose and Lewis a tetraose. Future studies could employ a wider substrate panel, including longer glycans with greater affinity for these carbohydrate-binding domains. Future studies could also incorporate the considerations discussed above while exploring the effects of tandemly positioning novel carbohydrate-binding domains in the engineering of α-L-fucosidases and other glycoside hydrolases to enhance enzyme activity across various applications.

## 4 Material and methods

**Interpro analysis of the α-L-fucosidases:** Domain architecture analysis of α-L-Fucosidases (PF01120; IPR057739) was performed using data downloaded from the Interpro database on 25 December 2025 [7].

**Structural analysis of the α-****L****-fucosidase constructs:** The three-dimensional structures of the protein constructs were predicted using the web-version of AlphaFold3 [9]. The ligand/substrate/product-complexed protein structures were also predicted using a local installation of Boltz2 [10]. The Boltz2 models were aligned with the crystal structure of *Thermotoga maritima* (PDB ID: 1ODU) using the cealign algorithm in PyMOL, and the L-fucose in 1ODU and the structural models were aligned using the pair-fit command.

**Cloning, expression, and purification of proteins:** *Sr*Fuc-pET28a(+) and *Sr*FucNaFLD-pET28a(+) were previously described [4] and used as such. The PCR amplified sequence coding for the N-terminal domain of MG1D1 (residues Y2 to G121 of Genbank QIQ49541.1), MN3α (residues N45 to A212 of Genbank QIQ49542.1), and MU1α (residues L36 to K182 of Genbank QIQ49543.1) were ligated in *Sr*Fuc-pET28a(+) at the Nco1 site to make the constructs MG1D1-*Sr*Fuc-pET28a(+), MN3α-*Sr*Fuc-pET28a(+), and MU1α-*Sr*Fuc-pET28a(+). Sanger DNA sequencing confirmed the nucleotide sequence of all the constructs. A mutation, A57D (with respect to the original MG1 clone), was noted in the MG1D1 region of the MG1D1-SrFuc clone.

The recombinant proteins were expressed and purified from a secondary culture of *E. coli* BL21(DE3) transformed with the appropriate plasmid construct, and cultured at 37 °C (200 rpm shaking) in Luria-Bertani broth. We induced recombinant protein expression with 1 mM IPTG at an OD600 of ∼0.6 and continued incubation at 37 °C for 4 h. The cells were collected by centrifugation and lysed with 300 mM sodium chloride (Merck), 20 mM Tris (hydroxymethyl) aminomethane (Merck), pH 7.5, 1% N-Laurylsarcosine sodium salt (Sigma) by ultrasonication (Material and Sonics INC) using 10 s pulses of amplitude 22% alternating with 10 s pulses of rest for 30 min. We clarified the lysate by centrifugation at 11000 RPM for 45 min. We purified the C-terminal hexahistidine-tagged recombinant proteins using a HisPur Ni-NTA resin (Thermo Fisher Scientific) column incubated in a Rotaspin (Tarson) for 3 h at 4 °C, then washed with 20 mM imidazole in 20 mM Tris(hydroxymethyl)aminomethane, pH 7.5, containing 300 mM sodium chloride. We eluted the recombinant protein using 200 mM imidazole in 20 mM Tris(hydroxymethyl)aminomethane, pH 7.5, containing 300 mM sodium chloride. The eluates were dialyzed using TBS (20 mM Tris and 150 mM NaCl) using a 10000 MWCO membrane. The protein concentration was determined from the OD_280_ measured on a DeNovix DS-11 spectrophotometer. We used Dynamic Light Scattering (DLS) measurements (Malvern) using the same standard proteins reported earlier [5] to estimate oligomeric status.

**α-L-Fucosidase Assays on 4-Methylumbelliferyl-α-L-fucopyranoside (4-MUF):** The α-L-fucosidase activities of the recombinant fucosidases were first measured on 4-methylumbelliferyl-α-L-fucopyranoside (4-MUF). The reaction setup consisted of a 70 μL reaction in TBS containing 10 mM calcium chloride, 0.3 μM α-L-fucosidase, and 10 μM 4-MUF (Sigma-Aldrich). The reactions were set up in a 96-well flat-bottom polypropylene microplate (Greiner Bio-One) at 37 °C, and the end product of the α-L-fucosidase reaction, i.e., a fluorescent product 4-methylumbelliferone (4-MU) (Ex: 325 nm, Em: 450 nm), was detected in kinetic mode at 1-min intervals for 6 hours (Cytation 5 multimode plate reader). A standard plot of 4-MU fluorescence at 2-fold dilutions from 10 μM was generated. Using the equation obtained by linear regression of this plot, the fluorescence measurements in the α-L-fucosidase assay were converted to 4-MU concentrations. The initial velocity of the α-L-fucosidase reaction was then determined by linear regression of the initial points of the progress curve. Using the same concentration of 0.3 μM for the α-L-fucosidase, we observed different specific activities for the different α-L-fucosidase constructs on 4-MUF. We subsequently adjusted the concentrations of the three biological replicates (independent protein preparations) for each protein construct used in the assay, ensuring similar activities with 4-MUF for effective comparison of the constructs with respect to their activity on natural fucosylated substrates.

In addition to measuring the initial velocities of the α-L-fucosidases by measuring the fluorescence of 4-MU, the L-fucose released in the reaction at the endpoint of 6 hours was also determined using an FDH- and diaphorase-coupled assay. The 70 μL α-L-fucosidase reaction mix was added to a 30 μL mixture containing 2 μM FDH (*Pseudomonas sp*. fucose dehydrogenase, purified as previously described [4], 17 μM resazurin (Sigma), 1.7 mM NADP (Sigma), 0.34 units/mL Diaphorase (Sigma), and 17 mM calcium chloride (Sigma). Resorufin fluorescence (λ_ex_: 530 nm; λem: 590 nm) was monitored in kinetic mode at 1-min intervals (Cytation 5 multimode plate reader) at 37 °C. The fluorescence readings obtained were plotted against time, and the slopes were used to determine the L-fucose formed at the endpoint (6 hours), using the standard curve described below.

The standard curve was generated using FDH initial velocities at different L-fucose concentrations (i.e., 70 μL L-fucose in 2-fold dilutions from 70 μM) added to a 30 μL reaction mixture containing the FDH assay mix. The initial velocities of FDH were determined by linear regression of the initial points from the resorufin fluorescence progress curve. The standard curve used the initial velocities of FDH calculated for different L-fucose dilutions, fit to a linear equation rather than the Michaelis-Menten equation because we used L-fucose concentrations lower than the *K*_*m*_ of FDH, considering the low L-fucose concentrations observed in the endpoint α-L-fucosidase reactions [4].

**α-L-Fucosidase Assays on natural fucooligosaccharide substrates:** The α-L-fucosidase activity of the recombinant constructs on the unlabelled natural fucosylated oligosaccharides - Lewis a tetrasaccharide (GLY054, Elicityl) and 2’ Fucosyllactose (GLY031, Elicityl) was determined similarly using the FDH- and Diaphorase-coupled endpoint assay. A 70 μL reaction mix was prepared by adding 1 mM oligosaccharide and recombinant α-L-fucosidase (at the concentrations mentioned above) to TBS containing 10 mM calcium chloride in a 96-well flat-bottom microplate (Greiner Bio-One), and incubated for 6 hours at 37 °C, following which the FDH mix was added, and the fluorescence of the resorufin formed was measured, as described above.

## Author contributions

T.N.C.R. conceived the study. S performed all the biochemical experiments and wrote the first draft of the manuscript. M.L. cloned the constructs reported in the study. S.S. performed the Boltz2 analysis and protein and ligand alignment. All authors participated in the critical review of the manuscript and approved the final version for communication.

## Funding

This work was funded by the Council of Scientific and Industrial Research, Government of India (CSIR-IMTECH Research Council-approved project OLP0178 to TNCR). S. and M.L. acknowledge the Council of Scientific and Industrial Research, Government of India, for their fellowship.

## Declaration of competing interest

The authors declare that they have no known competing financial interests or personal relationships that could have appeared to influence the work reported in this paper.

## Data Availability

Data associated with the plots presented in the figures are available as Supplementary datasets. Other data will be made available upon reasonable request.
