# Supplementary material for: Exploring the effect of tandem non-native non-F-type lectin carbohydrate-binding domains on Streptosporangium roseum α-L-Fucosidase activity
Source: Biochem Biophys Rep. 2026 Sep 15;48:102797. doi: 10.1016/j.bbrep.2026.102797 (PMC13594953; doi:10.1016/j.bbrep.2026.102797)
Supplement: Supplementary file 1 [file mmc1.pdf]

Supplementary Figures S1 to S5

Exploring the effect of tandem non-native non-F-type lectin carbohydrate-binding domains on *Streptosporangium roseum* α-L-Fucosidase activity

Shivangi[1,2], Madhu Lata[1,2], Srikrishna Subramanian[1,2], T.N.C. Ramya[1,2,*]

[1]CSIR- Institute of Microbial Technology, Sector 39-A, Chandigarh 160036, INDIA

[2]Academy of Scientific & Innovative Research (AcSIR), Ghaziabad, Uttar Pradesh 201002, India.

*Correspondence to be addressed to T.N.C. Ramya, CSIR- Institute of Microbial Technology, Sector 39-A, Chandigarh 160036, INDIA. Tel: 91-172-2880243; E-mail: ramya.tnc@csir.res.in

Running Title: Tandem carbohydrate binding domains and alpha-L-fucosidase

Keywords: α-L-fucosidase, carbohydrate-binding domain, CBM, lectin, tandem domain

Supplementary Information:

A separate .xlsx file with Supplementary Tables S1 to S8 and a Supplementary Data .xlsx file containing the raw data behind all the plots in the figures of this manuscript also accompany this manuscript.

Figure S1

A. MG1D1-*Sr*Fuc

MAYYPVKFKSGNYLSDKGAGNKMQTVAQKGDATMFQFIGTQSSFIMKSKSGNYVGF**D**NERFITTTKEN
AVKMSIVNGSAEKYYELQRVGQTKRMNQFGGTGVGVQLGEWNAGDNNNQLFFEGTMDTPPVYEPTV
ESLNSHPVPQWFNDDKFGIFIHWGAYSVPAWGPRGSYAEWYGNYMNAGGSATNAHHKATYGQDFNY
DAFLQQWKAEKFDPADWVKLFKDAGAKYFVLTSKHHEGMALWDSKSSGRDSVDLGPGRDLAKELFDA
ARKDEAKLKAGFYYSLYEWYNPAYTGRPATNPYTGAEIPYTGAPGGGDYVKDYMLPQMRELIDGYDPDI
IWCDGQWEKPASYWNTAGVIADYYNKALNSGKEVAVANRCKIQSGNLDSPELDFQTPEYTVKPDIDPVK
WESSRGIAHSYGYNQNEPEEDHLTSDQLVDSLVDIVSKNGNLLLDIGPKADGTIPEIQRQRLLDIGAWLK
ANGEAIYGTTYWNRAEEKGGPDDVRYTAKDATLYATALKWPGEQLTLGADLPVDHSTRITMLGSGARLS
WSRDEQGRVVVNTPQEAGKHAYVFKIETPGLEHHHHHH

B. MN3α-*Sr*Fuc

MANDRVLSSFSLQQIGKDKQTVNVFASRAAYEDHTSSVFNVEAGSELTASFNYSGQWMNGYIYIDRD
NDGEFSYKEGQWDQAGTDLVAYSFYTTLANPKVNDGNGYNSTGASVSGNDRSNVNPPSFTAPTEP
GTYRIRFKIDWNCILPGGSSDILSDGGGVWDATLNVVATMDTPPVYEPTVESLNSHPVPQWFNDDKF
GIFIHWGAYSVPAWGPRGSYAEWYGNYMNAGGSATNAHHKATYGQDFNYDAFLQQWKAEKFDPAD
WVKLFKDAGAKYFVLTSKHHEGMALWDSKSSGRDSVDLGPGRDLAKELFDAARKDEAKLKAGFYYS
LYEWYNPAYTGRPATNPYTGAEIPYTGAPGGGDYVKDYMLPQMRELIDGYDPDIIWCDGQWEKPASY
WNTAGVIADYYNKALNSGKEVAVANRCKIQSGNLDSPELDFQTPEYTVKPDIDPVKWESSRGIAHSY
GYNQNEPEEDHLTSDQLVDSLVDIVSKNGNLLLDIGPKADGTIPEIQRQRLLDIGAWLKANGEAIYGTT
YWNRAEEKGGPDDVRYTAKDATLYATALKWPGEQLTLGADLPVDHSTRITMLGSGARLSWSRDEQG
RVVVNTPQEAGKHAYVFKIE**TPGLEHHHHHH**

C. MU1α-*Sr*Fuc

MALFCKENYVADGTGFEWDINVDWDTQKIVMSIDPSTCTGSCEDVIGLGAVPTAWDNTLHLYRTSADQ
MLQGYFNVPGGSNNNTDKFAETAPFLVEVSKAQGFVVNNEVKIASSAMSYLFTLSTVKMGTGEGANDK
SRATYNYVKVVDKTMDTPPVYEPTVESLNSHPVPQWFNDDKFGIFIHWGAYSVPAWGPRGSYAEWYG
NYMNAGGSATNAHHKATYGQDFNYDAFLQQWKAEKFDPADWVKLFKDAGAKYFVLTSKHHEGMALW
DSKSSGRDSVDLGPGRDLAKELFDAARKDEAKLKAGFYYSLYEWYNPAYTGRPATNPYTGAEIPYTGA
PGGGDYVKDYMLPQMRELIDGYDPDIIWCDGQWEKPASYWNTAGVIADYYNKALNSGKEVAVANRCKI
QSGNLDSPELDFQTPEYTVKPDIDPVKWESSRGIAHSYGYNQNEPEEDHLTSDQLVDSLVDIVSKNGNL
LLDIGPKADGTIPEIQRQRLLDIGAWLKANGEAIYGTTYWNRAEEKGGPDDVRYTAKDATLYATALKWPG
EQLTLGADLPVDHSTRITMLGSGARLSWSRDEQGRVVVNTPQEAGKHAYVFKIETPGLEHHHHHH

Supplementary figure S1: Confirmation of cloned constructs by Sanger sequencing. (**A**) MG1D1-*Sr*Fuc. (**B**) MN3α-*Sr*Fuc. (**C**) MU1α-*Sr*Fuc. DNA sequencing coverage is indicated by underlining. The yellow highlight indicates a mutation in the amino acid sequence with respect to the original MG1 clone (A57D). T7 forward and reverse primers were used for sequencing.

Figure S2

A. MG1D1-*Sr*Fuc

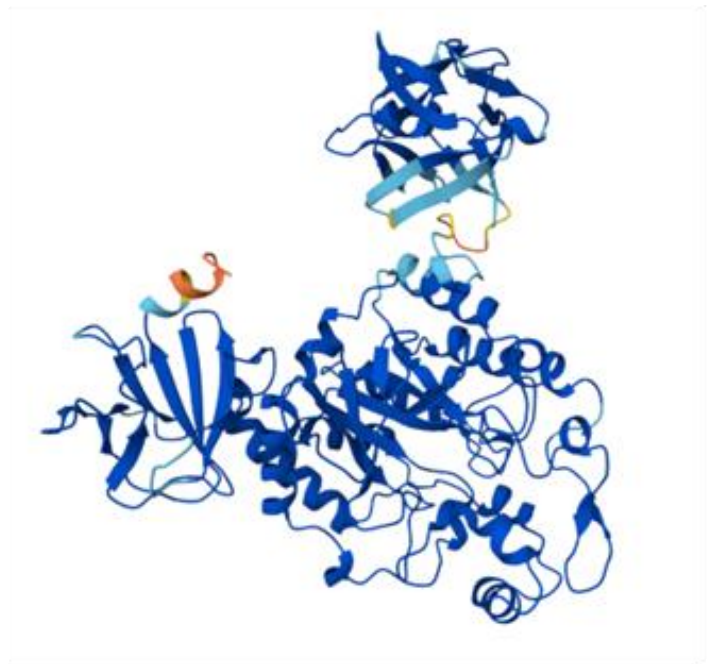

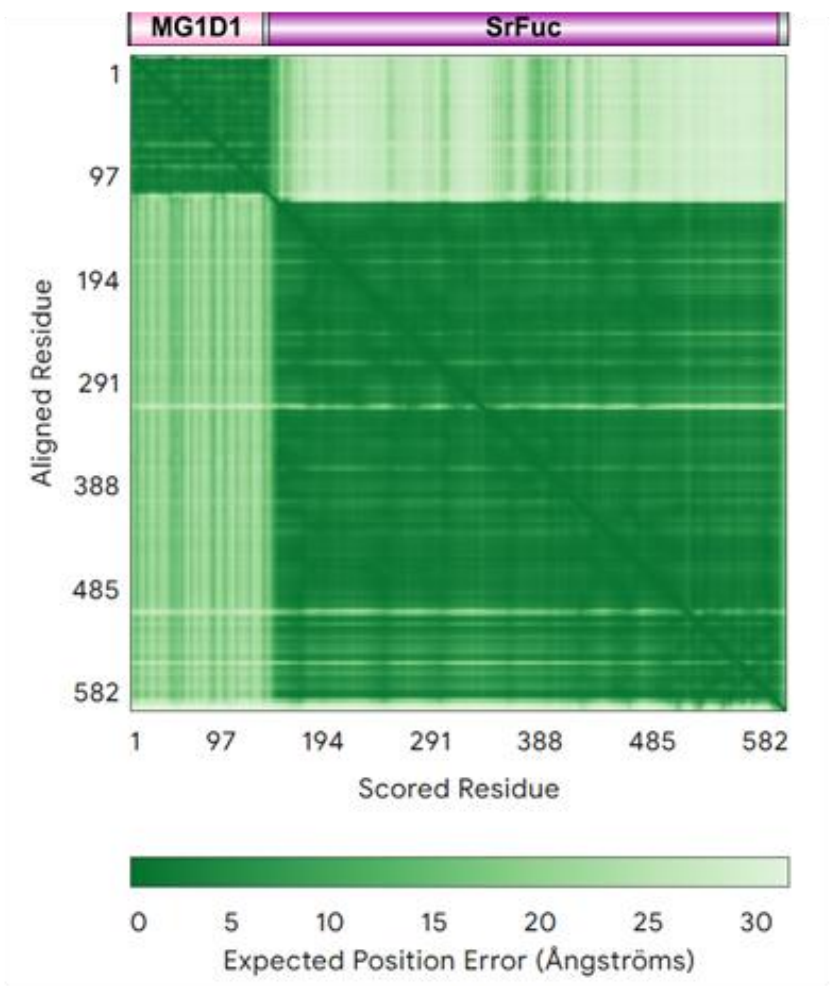

Average plDDT score = 91.62.
pTM = 0.8

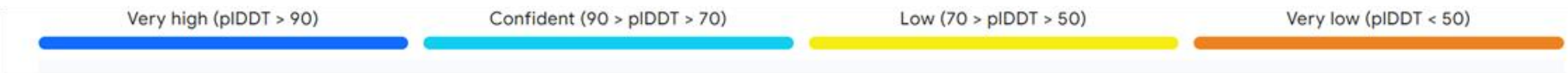

B. MN3α-SrFuc

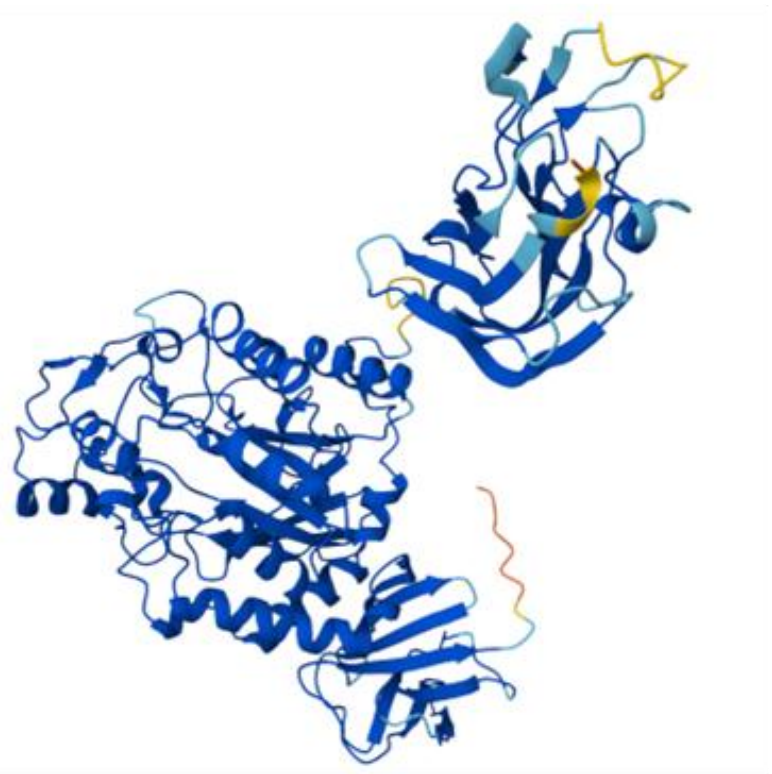

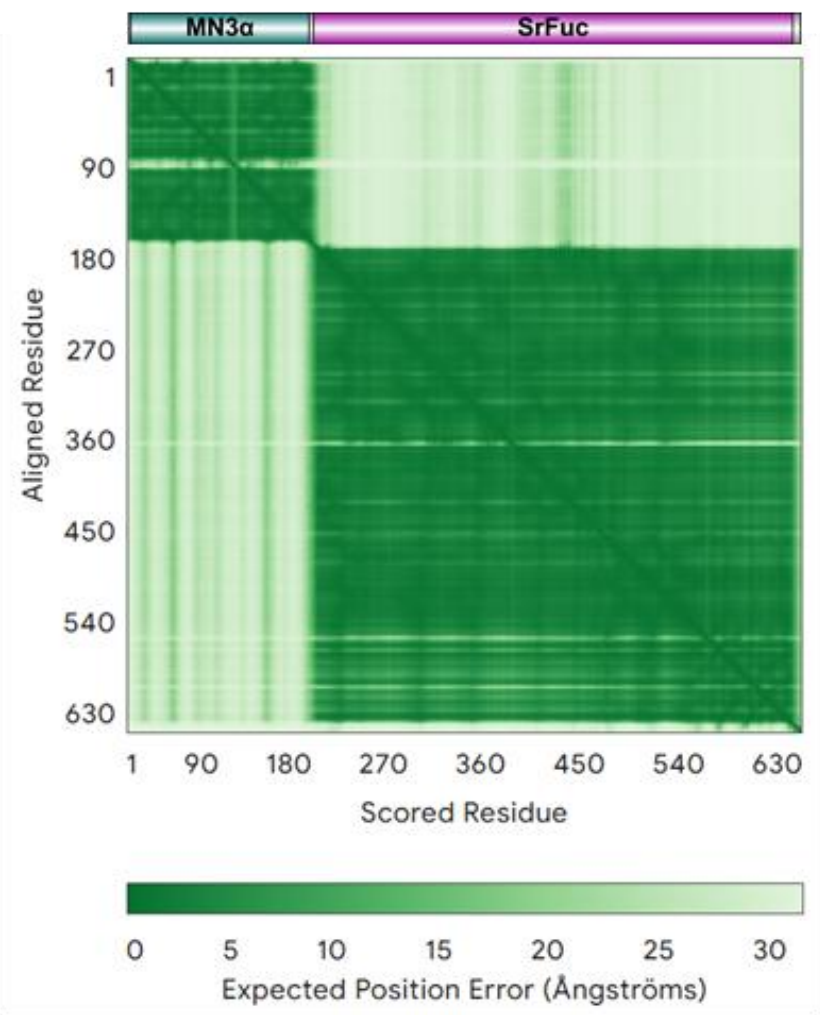

Average plDDT score = 91.61
pTM = 0.73

C. MU1α-*Sr*Fuc

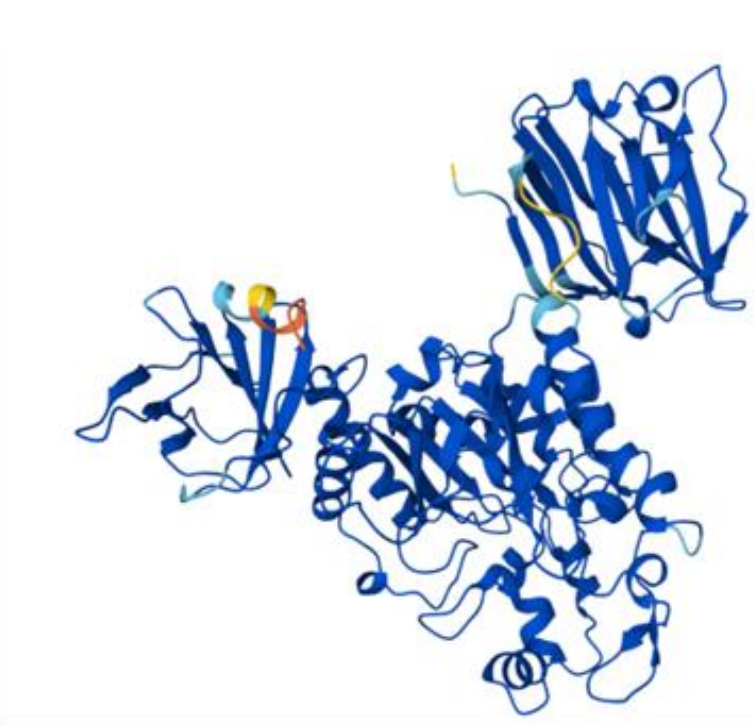

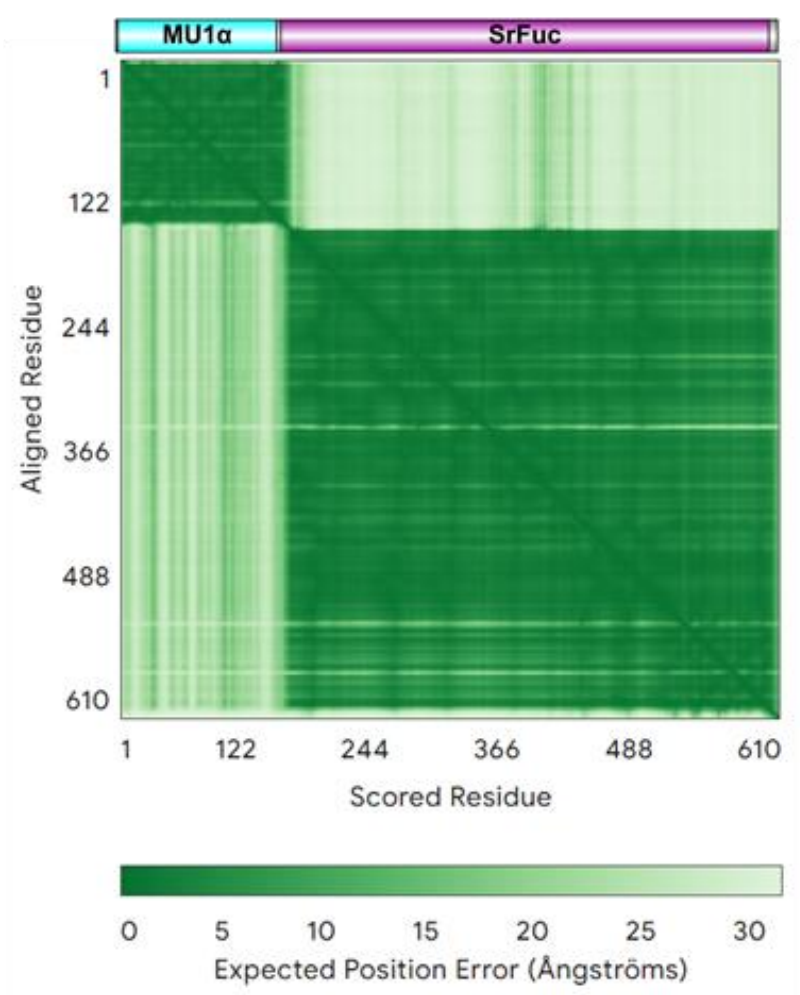

Average plDDT score = 93.00
pTM = 0.77

D. MG1D1-SrFuc_LFuc

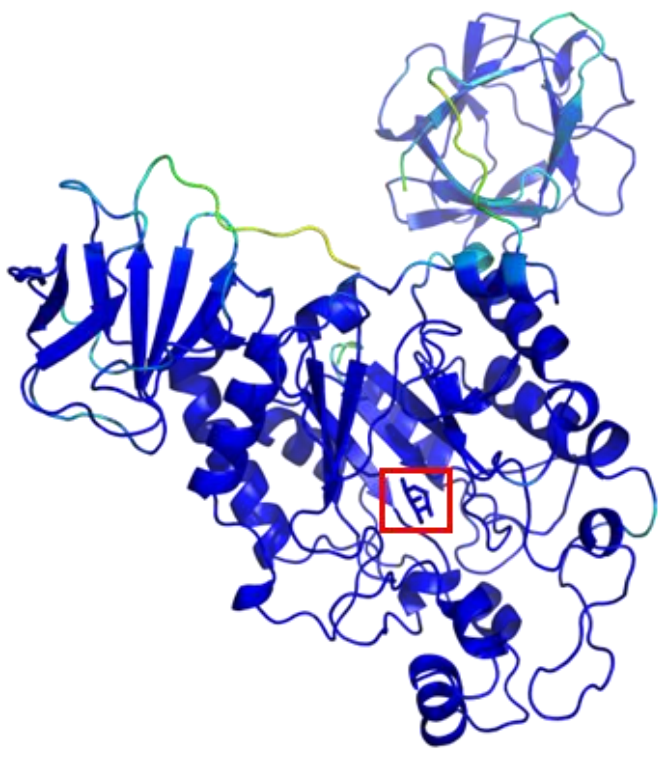

E. MN3α-SrFuc_LFuc

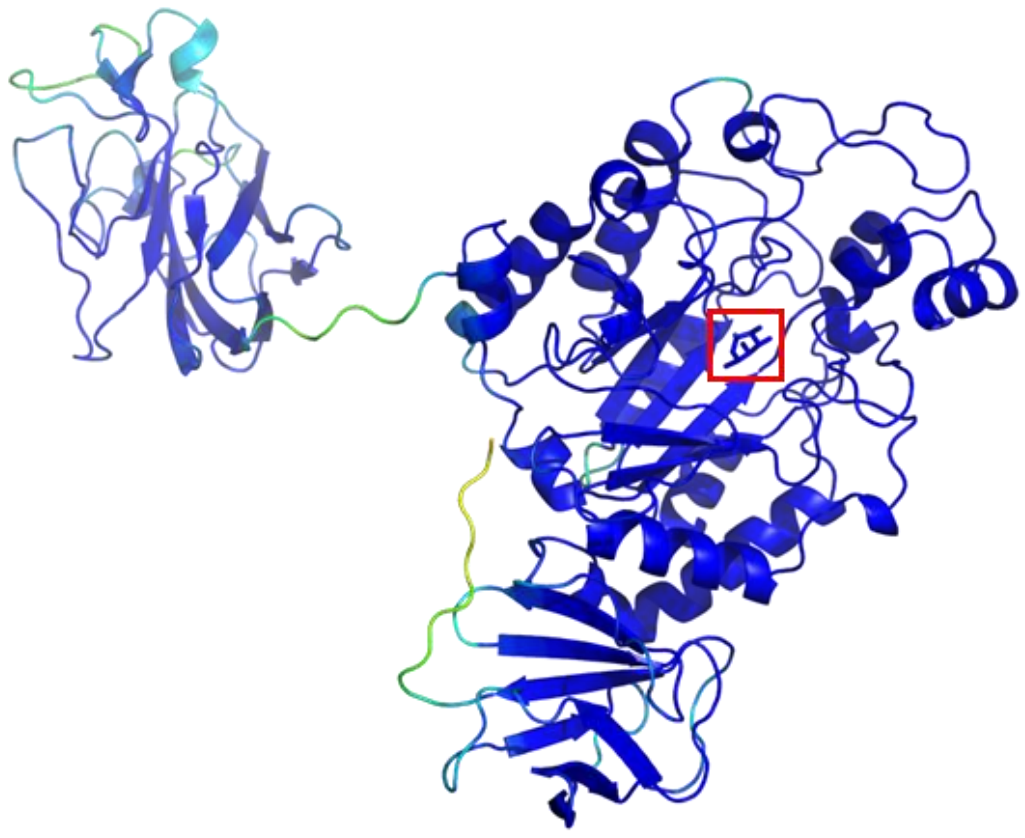

F. MU1α-SrFuc_LFuc

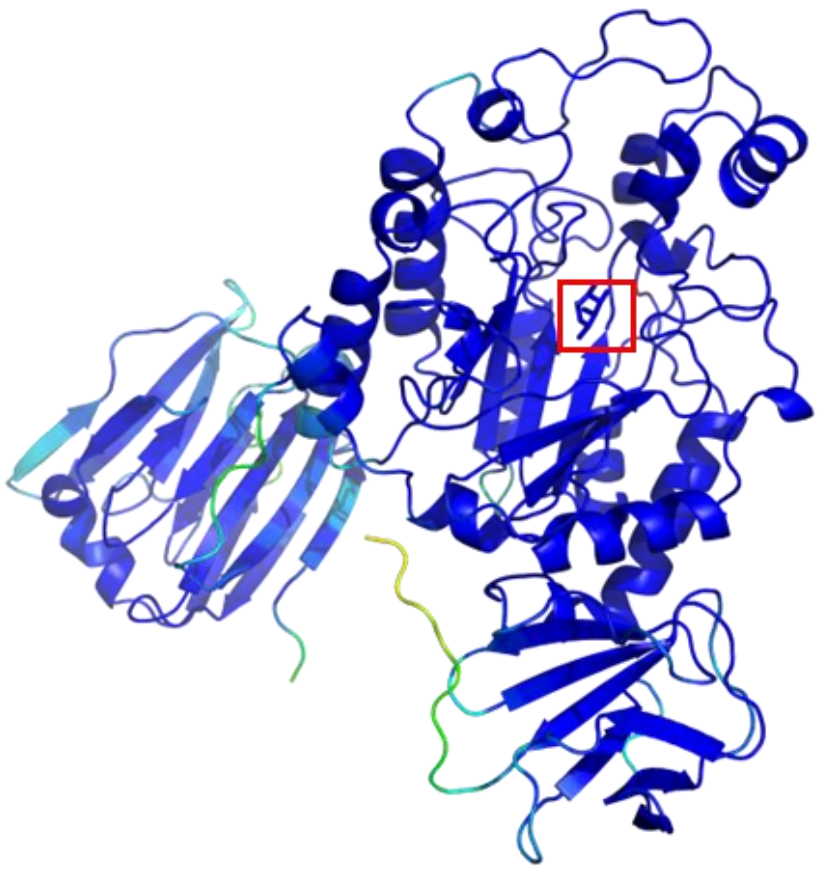

G. SrFLD_LFuc

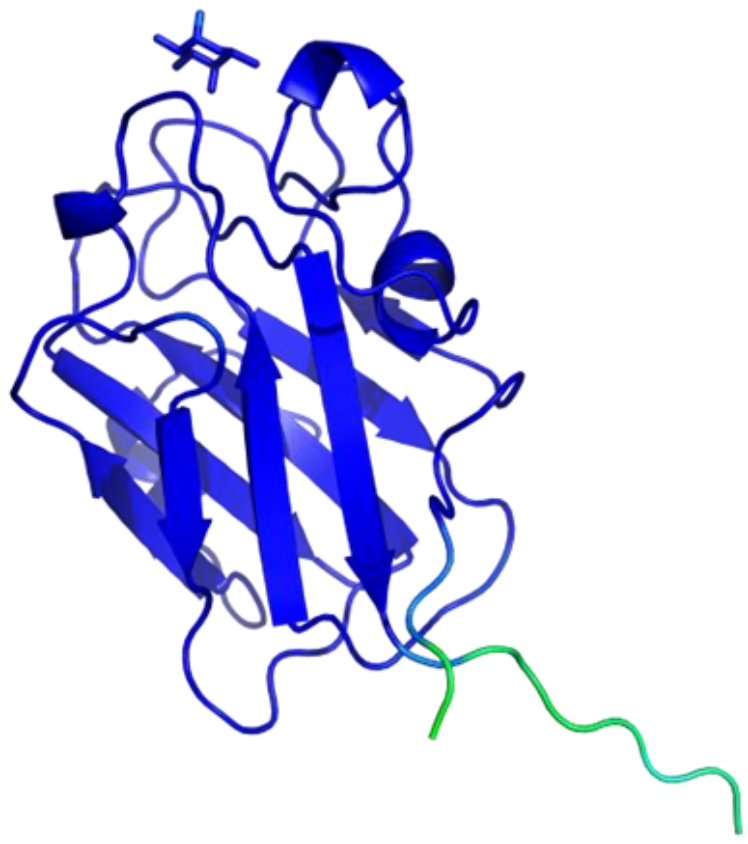

H. SrFuc_LFuc

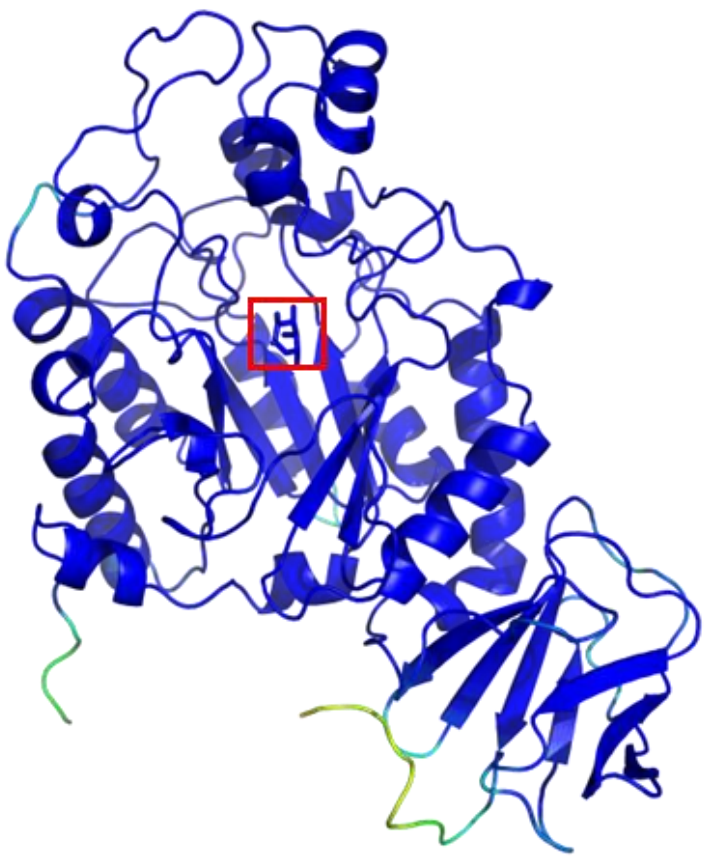

I. SrFucNaFLD_LFuc

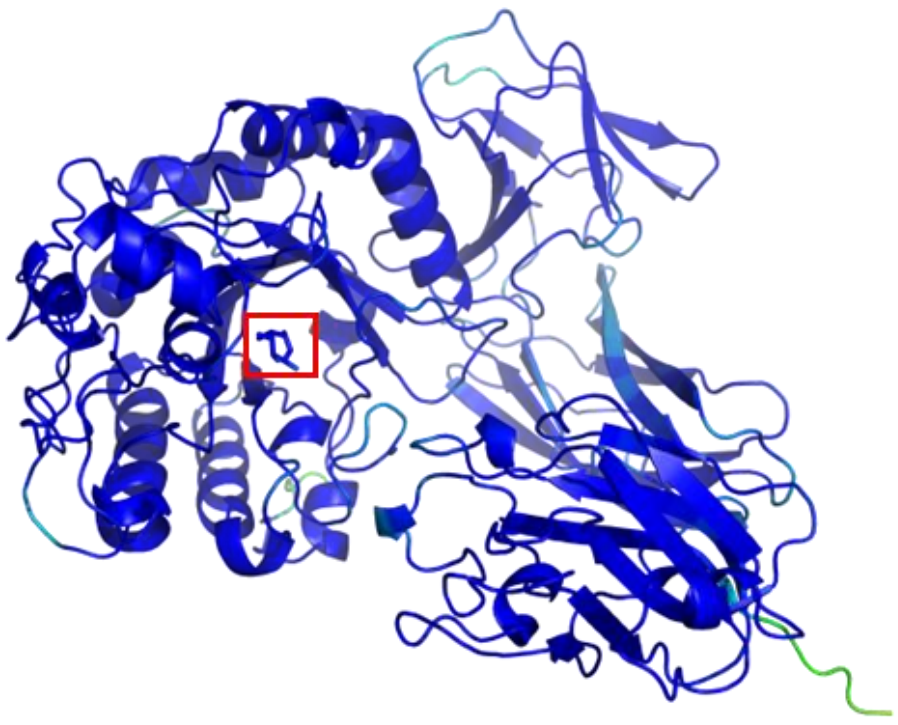

J. MG1D1_LFuc

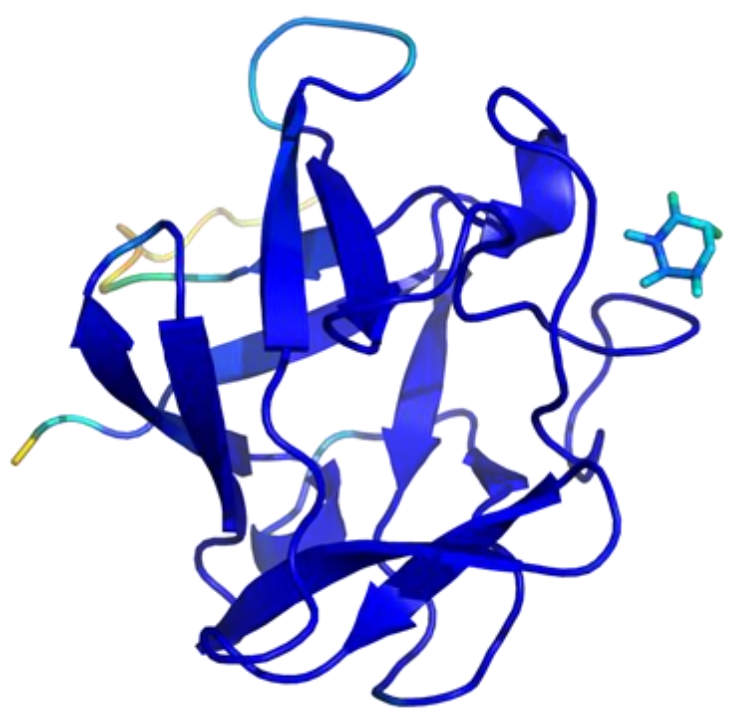

Supplementary figure S2: AlphaFold3-predicted protein models and Boltz2-predicted L-fucose-complexed protein models. (**A-C**) AlphaFold3-predicted protein models of (**A**) MG1D1-*Sr*Fuc. (**B**) MN3α-*Sr*Fuc. (**C**) MU1α-*Sr*Fuc. The AlphaFold3-predicted structures are colored using a pLDDT score-based rainbow scheme, with dark blue indicating high pLDDT scores and red indicating low pLDDT scores. The pTM and the average pLDDT score are provided below the model. The PAE plot is provided to the right of the structural model and is accompanied by the domain architecture directly above it to provide domain context. (**D-J**) Botlz2-predicted structural models of L-fucose-complexed (**D**) MG1D1-*Sr*Fuc. (**E**) MN3α-*Sr*Fuc. (**F**) MU1α-*Sr*Fuc. (**G**) *Sr*FLD. (**H**) *Sr*Fuc. (**I**) *Sr*FucNaFLD. (**J**) MG1D1-*Sr*Fuc. The Boltz2-predicted models are colored using a pLDDT score-based rainbow scheme, with dark blue indicating high pLDDT scores and red indicating low pLDDT scores. The ligand is boxed in red for easy visualization, where required.

Figure S3

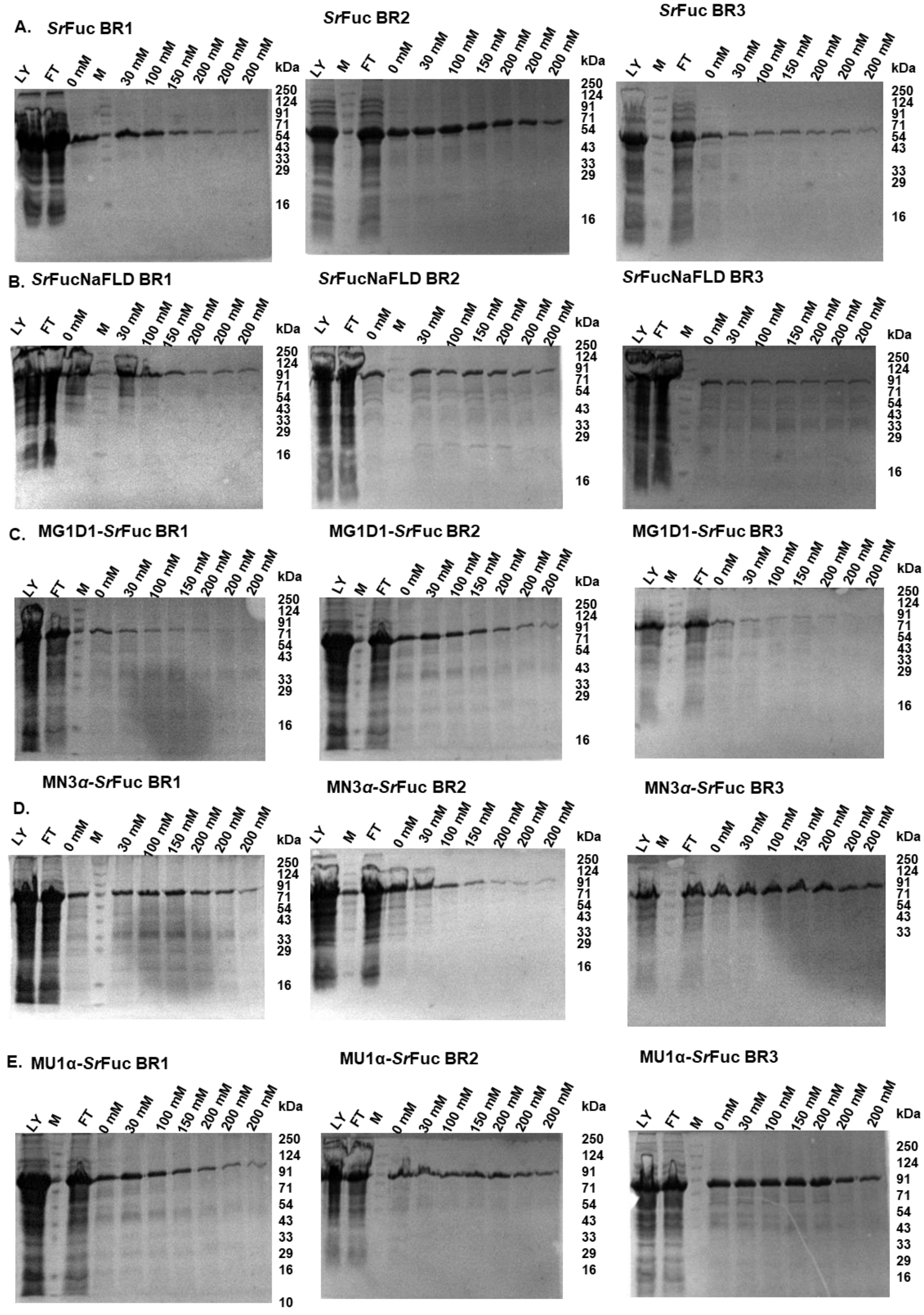

A. SrFuc BR1
LY FT 0 mM M 30 mM 100 mM 150 mM 200 mM 200 mM 200 mM
kDa
250 124 91 71 54 43 33 29 16
SrFuc BR2
LY M FT 0 mM 30 mM 100 mM 150 mM 200 mM 200 mM 200 mM
kDa
250 124 91 71 54 43 33 29 16
SrFuc BR3
LY M FT 0 mM 30 mM 100 mM 150 mM 200 mM 200 mM 200 mM
kDa
250 124 91 71 54 43 33 29 16
B. SrFucNaFLD BR1
LY FT 0 mM M 30 mM 100 mM 150 mM 200 mM 200 mM 200 mM
kDa
250 124 91 71 54 43 33 29 16
SrFucNaFLD BR2
LY FT 0 mM M 30 mM 100 mM 150 mM 200 mM 200 mM 200 mM
kDa
250 124 91 71 54 43 33 29 16
SrFucNaFLD BR3
LY FT M 0 mM 30 mM 100 mM 150 mM 200 mM 200 mM 200 mM
kDa
250 124 91 71 54 43 33 29 16
C. MG1D1-SrFuc BR1
LY FT M 0 mM 30 mM 100 mM 150 mM 200 mM 200 mM 200 mM
kDa
250 124 91 71 54 43 33 29 16
MG1D1-SrFuc BR2
LY M FT 0 mM 30 mM 100 mM 150 mM 200 mM 200 mM 200 mM
kDa
250 124 91 71 54 43 33 29 16
MG1D1-SrFuc BR3
LY M FT 0 mM 30 mM 100 mM 150 mM 200 mM 200 mM 200 mM
kDa
250 124 91 71 54 43 33 29 16
D. MN3α-SrFuc BR1
LY FT 0 mM M 30 mM 100 mM 150 mM 200 mM 200 mM 200 mM
kDa
250 124 91 71 54 43 33 29 16
MN3α-SrFuc BR2
LY M FT 0 mM 30 mM 100 mM 150 mM 200 mM 200 mM 200 mM
kDa
250 124 91 71 54 43 33 29 16
MN3α-SrFuc BR3
LY M FT 0 mM 30 mM 100 mM 150 mM 200 mM 200 mM 200 mM
kDa
250 124 91 71 54 43 33
E. MU1α-SrFuc BR1
LY M FT 0 mM 30 mM 100 mM 150 mM 200 mM 200 mM 200 mM
kDa
250 124 91 71 54 43 33 29 16 10
MU1α-SrFuc BR2
LY FT M 0 mM 30 mM 100 mM 150 mM 200 mM 200 mM 200 mM
kDa
250 124 91 71 54 43 33 29 16
MU1α-SrFuc BR3
LY FT M 0 mM 30 mM 100 mM 150 mM 200 mM 200 mM 200 mM
kDa
250 124 91 71 54 43 33 29 16

F.

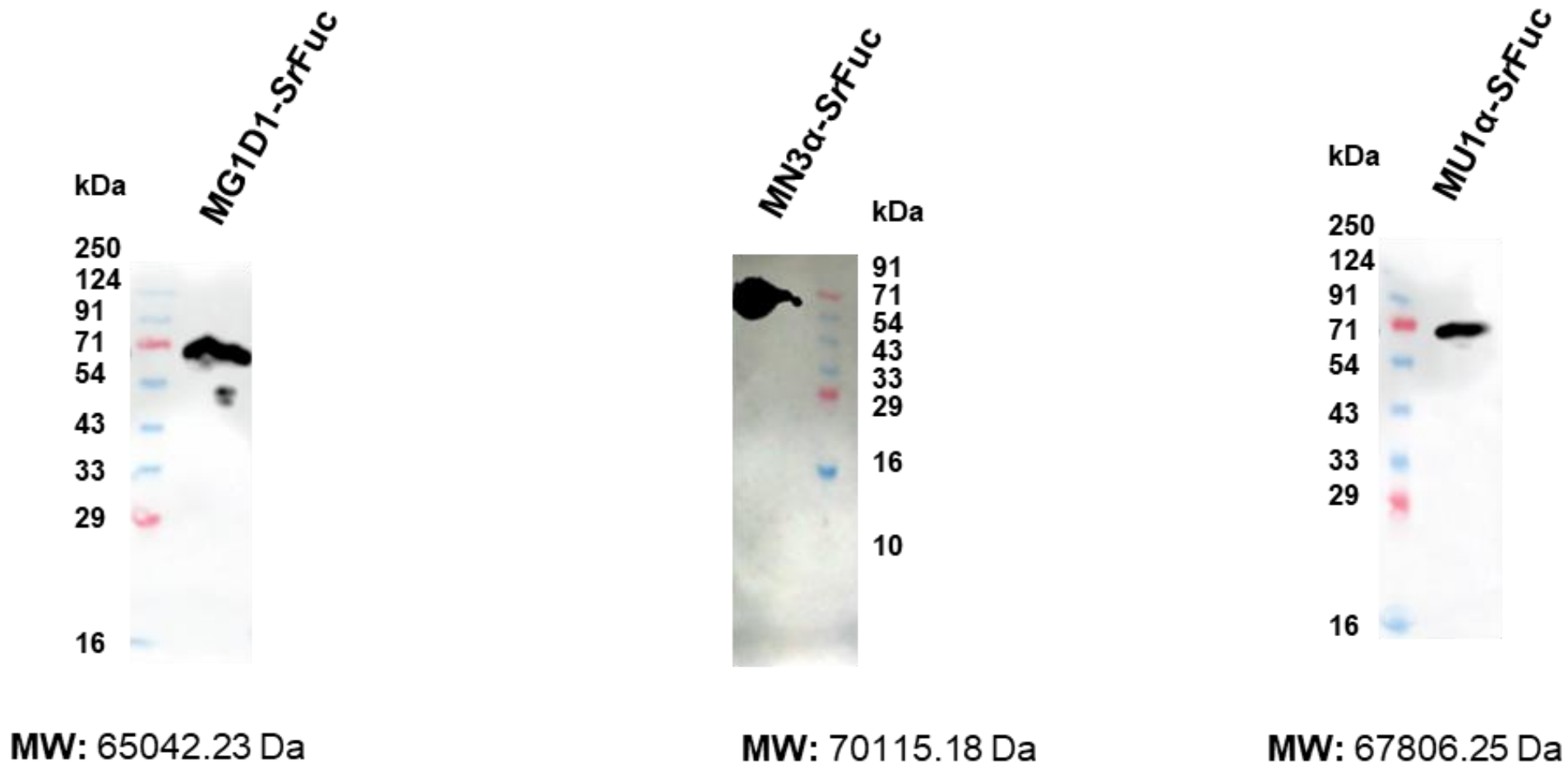

Supplementary figure S3: Purification of recombinant proteins. (**A-E**) Coomassie blue-stained SDS-PAGE gel showing recombinant protein purification by nickel metal ion affinity chromatography. (**A**) Three biological replicates (independent protein preparations) of SrFuc. (**B**) Three biological replicates (independent protein preparations) of *Sr*FucNaFLD. (**C**) Three biological replicates (independent protein preparations) of MN3α-*Sr*Fuc. (**D**) Three biological replicates (independent protein preparations) of MG1D1-*Sr*Fuc. (**E**) Three biological replicates (independent protein preparations) of MU1α-*Sr*Fuc. LY: lysate, FT: Flow through, M: Protein molecular mass marker. 0 mM, 30 mM, 100 mM, 150 mM, and 200 mM refer to column eluates with the corresponding imidazole concentrations. Pure fractions of protein from the 200 mM imidazole eluates were pooled together, and the resulting solution was dialyzed against 20 mM Tris-buffered saline (150 mM NaCl) and used for the experiments reported in this study. (**F**) Western blot probed with anti-His tag antibody to confirm the recombinant expression of 6XHis-tagged constructs MG1D1-*Sr*Fuc, MN3α-*Sr*Fuc, and MU1α-*Sr*Fuc.

Figure S4

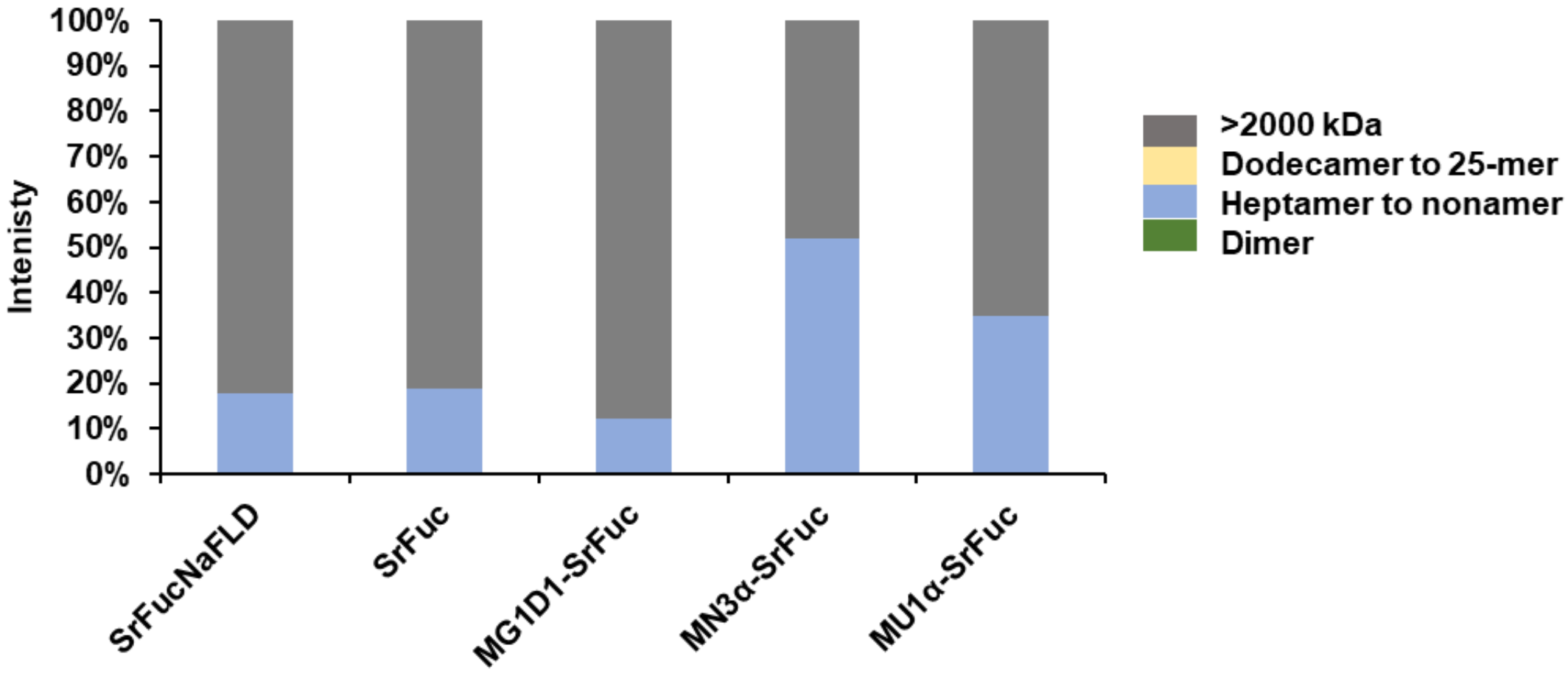

Supplementary figure S4: Oligomeric status of the recombinant protein constructs as measured by DLS. Oligomeric profiles of the recombinant protein constructs, as obtained by conversion of the particle sizes measured by DLS to the order of oligomerization (number of monomeric units in the oligomer), taking into account theoretical molecular masses for each construct.

Figure S5

A.

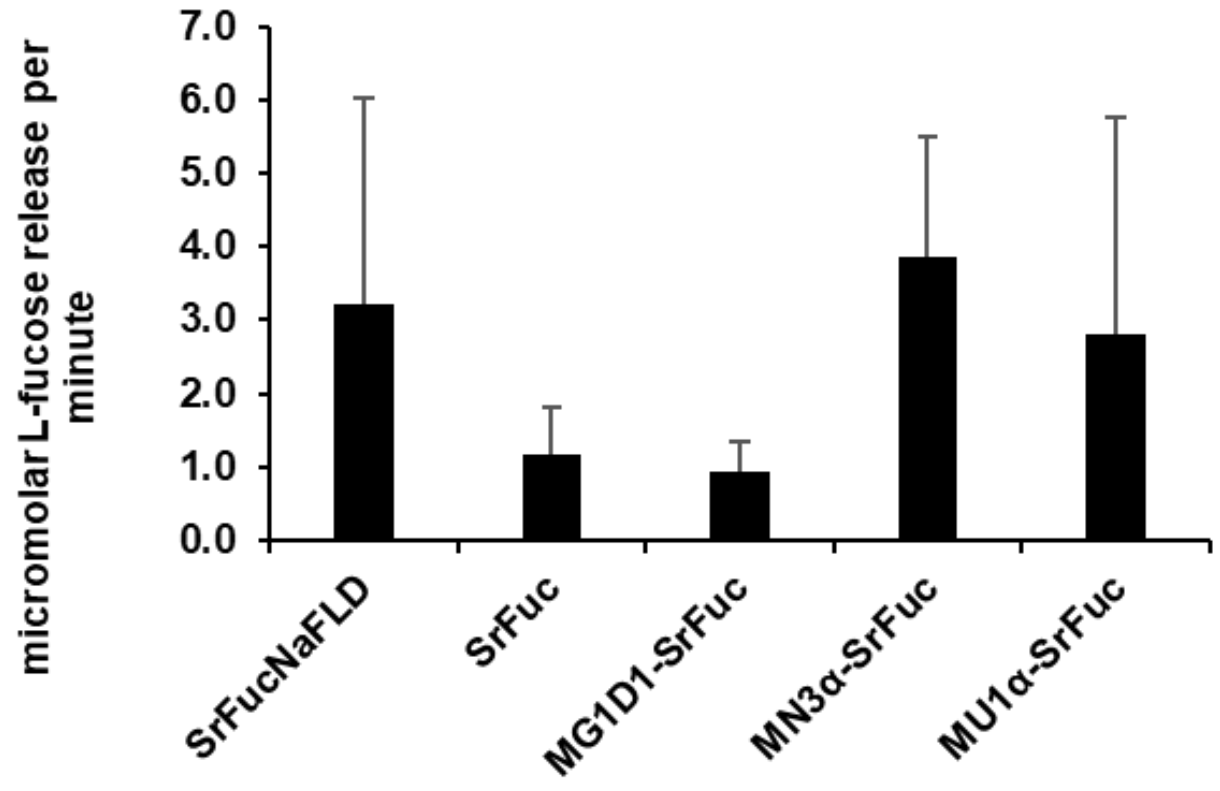

B.

Protein Name	Concentration of purified protein stock (μM)	Volume of purified enzyme added in the 100 μL 4-MUF reaction mix for a final 0.3 μM enzyme concentration (μL)	Volume of purified enzyme added in the 100 μL 4-MUF reaction mix for normalized L-fucose release from 4-MUF (μL)	Concentration of enzyme in the 100 μL 4-MUF reaction mix for normalized L-fucose release from 4-MUF (μM)
SrFucNaFLD BR1	0.7	43	17	0.1
SrFucNaFLD BR2	3	10	29	0.9
SrFucNaFLD BR3	0.8	38	7	0.1
SrFuc BR1	0.85	35	13	0.1
SrFuc BR2	2	15	24	0.5
SrFuc BR3	1.4	14	11	0.2
MG1D1-SrFuc BR1	1	30	22	0.2
MG1D1-SrFuc BR2	1	30	44	0.4
MG1D1-SrFuc BR3	1	30	22	0.2
MN3α-SrFuc BR1	2.3	13	4.3	0.1
MN3α-SrFuc BR2	0.9	34	19	0.2
MN3α-SrFuc BR3	2.9	11	3.7	0.1
MU1α-SrFuc BR1	1.8	17	17	0.3
MU1α-SrFuc BR2	1.8	17	5.5	0.1
MU1α-SrFuc BR3	1.5	20	53	0.8

Supplementary figure S5: Enzyme activity of α-L-fucosidase constructs on the 4-MUF substrate. (**A**) α-L-fucosidase activity on 4-MUF. Initial velocities (μM L-fucose formed per minute) are plotted for reactions containing 0.3 μM α-L-fucosidase and 10 μM 4-MUF. (**B**) Tabulation of reaction parameters for normalization of α-L-fucosidase activity in a 100 μL reaction with 4-MUF.
